# Optimization of Parameters and Comparison of Detection Signals for Planar Coil Particle Detection Sensors with Different Core Materials

**DOI:** 10.3390/mi15121520

**Published:** 2024-12-20

**Authors:** Changzhi Gu, Chao Liu, Bo Liu, Wenbo Zhang, Chenzhao Bai, Chenyong Wang, Yuqing Sun, Hongpeng Zhang

**Affiliations:** 1 Marine Engineering College, Dalian Maritime University, Dalian 116026, China; msagcz@163.com (C.G.); chaoliu@dlmu.edu.cn (C.L.); 13674137039@163.com (B.L.); zhangwenbo@dlmu.edu.cn (W.Z.); baichenz@163.com (C.B.); wangcy_dlmu@163.com (C.W.); sunyq@dlmu.edu.cn (Y.S.); 2 State Key Laboratory of Maritime Technology and Safety, Dalian 116026, China

**Keywords:** inductance detection, magnetic field optimization, parameter optimization

## Abstract

The cleanliness of lubricating oil plays a key role in determining the operational health of mechanical systems, serving as a critical metric that delineates the extent of equipment wear. In this study, we present a magnetic-core-type planar coil particle detection sensor. The detection accuracy and detection limit are improved by optimizing the magnetic field inside the sensor. The optimization of the magnetic field is achieved through the finite element simulation analysis of the coil and the magnetic core. First, the finite element simulation software COMSOL 6.0 is used to model the sensor in three dimensions (3D). Then, we study the distribution of the magnetic field under different coil radii, core conductivity levels, and other parameters. We obtain the sensor structure after optimizing the magnetic field. The sensor is made using experimental methods, and the iron particles and copper particles are detected. The results show that the lower limit of detection of iron particles can reach 46 μm, and the lower limit of detection of copper particles can reach 110 μm.

## 1. Introduction

In the field of industrial machinery, oil is widely used as an energy transfer medium in industrial production; lubricants are also widely used. In hydraulic systems and lubrication systems, solid particles are the main pollutants. More than 75% of hydraulic systems failures, about 35% of diesel engine operating faults, 38.5% of gear failures, and 40% of rolling bearing failures are due to the contamination caused by oil. Therefore, keeping the machine fluid clean is important to improving the stability of a mechanical system as well as to extending its working life [1,2].

Particulate contaminants in oil fluids can be categorized into three major types: gas, liquid, and solid [3,4,5]. When detecting these contaminants, they can be categorized using traditional ferro-spectroscopic detection [6,7,8,9,10]. Additionally, they can be detected using spectroscopic detection [11], and acoustic, optical, and electromagnetic particle counting in combination with microfluidic detection technologies [12,13,14,15,16,17]. Among these techniques, the combination of inductive and microfluidic detection technology has become a hot research topic in the last decade [18].

In the last ten years, many universities and research institutions have conducted research on inductive particle sensors, covering areas such as the basic theory of sensors, sensor structure design, circuit design, and signal resolution extraction technology [19,20,21]. The optimization of sensor structure design is mainly achieved through the optimal design of coils and magnetic cores, and detection accuracy is improved by controlling the local magnetic field distribution in the detection area. Jiang et al. proposed a high-throughput inductive pulse sensor based on the inductive Coulter counting principle, which uses a single-plane coil structure to detect metallic abrasive particles in lubricating oils; it can detect ferromagnetic abrasive particles up to 50 μm and non-ferromagnetic abrasive particles up to 150 μm [22]. MuThuvel et al. proposed a new passive wireless LC sensing method based on an abrasive grain monitoring sensor [23]. The sensor can monitor abrasive metallic particles of more than 25 μm in a pipe with an inner diameter of 25.4 mm and an outer diameter of 80 mm, and can be applied to pipes with large diameters and thicknesses. Ishinbaev et al. established a physical model of the motion of metal particles in an electromagnetic sensor and simulated and analyzed the relationship between the amplitude of the output signal and the parameters of the coil and metal particles in the system; this provided a theoretical basis for the electromagnetic-type abrasive particle detection method [24]. Wang et al. investigated the aliasing signals generated when a large number of abrasive particles pass through an inductive-type sensor; they found that the induced voltage is proportional to the abrasive particle velocity and the magnetic induction strength, and the peak-to-peak value of the induced electromotive force is basically proportional to the radius of the abrasive particles instead of the volume of abrasive particles [25]. Zhu et al. proposed a sensor structure consisting of a sensor with a 3 × 3 sensing channel structure; they tested the sensor on a lubricant fluid sample with a flow rate of 460 mL/min, showing that the sensitivity of the sensor in detecting ferromagnetic abrasive particles could reach 50 μm [19,20,21,26].

Previous studies have not systematically investigated the influence of different parameters of the coil and magnetic core on the magnetic field in the process of optimizing the design of sensor structure parameters. Therefore, this paper investigates the effects of the parameters of the coil and magnetic core on the magnetic field distribution and particle detection signal using finite element simulation analysis and experiments, which is of great significance for the optimal design of the sensor structure.

## 2. Sensor Design

**Figure 1:**
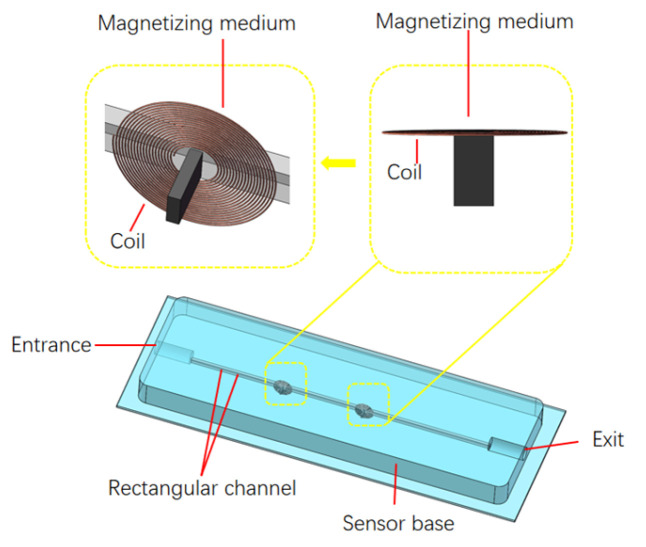
The coil’s magnetic core sensor.

We designed a planar coil magnetic core detection sensor, as shown in Figure 1. Its main components are a channel entrance, a rectangular channel, a detection unit, and a channel exit. The detection unit comprises a planar coil placed parallel to the channel. A magnetic core is placed in the center of the coil. The coil has an inner diameter of 900 µm, and each layer has 20 turns, with a total of 3 layers. The thickness of the magnetic core is 300 µm, the width is 800 µm, and the length is 1.5 mm. The rectangular channel passes between the coils and is directly opposite the magnetic core and close to the coil, with a width of 500 µm and a height of 900 µm. The rectangular channel design can make full use of the sensing area between the coils to ensure the sensor’s detection flux.

The sensor has a cuboid structure, and it is cast using polydimethylsiloxane (PDMS), which makes it easy to embed the detection unit, flow path, and magnetic core. As a non-conductive material, PDMS shows low electromagnetic loss in electromagnetic applications. This means that, when PDMS is used as the medium in the inductor coil, the signal attenuation can be effectively reduced, and the sensitivity of the sensor and the signal’s transmission efficiency can be improved. PDMS has a stable dielectric constant, which is very important to maintaining the consistency and predictability of the electromagnetic field. The stable dielectric characteristics ensure the performance consistency of the sensor at different frequencies, which helps to improve the accuracy and repeatability of the detection. They can also play a role in suppressing electromagnetic interference. The use of PDMS in inductor particle detection sensors can reduce interference from external electromagnetic fields, thereby improving the stability and reliability of the sensor. In addition, the plasticity and micromachining compatibility of PDMS facilitate the design and manufacture of inductors with complex shapes and micro-structures, and this design flexibility can be used to optimize the sensor’s response to specific-frequency electromagnetic fields, further improving the sensor’s performance and specificity. The chemical and physical stability of PDMS enables it to maintain the same performance under various environmental conditions (such as temperature and humidity changes), and it has a natural advantage in maintaining the stable propagation of electromagnetic fields.

The sensor channel is designed as a rectangular channel structure, considering that the circular hole channel has poor uniformity in the distribution of the magnetic field; moreover, under the condition of the same flux, the circular channel has a longer length along the coil axis, and the magnetic induction intensity at the center of the flow channel is weaker than that of the rectangular channel under the same flux. The channel inlet and outlet are designed at the edge of the sensor at both ends of the channel. The inlet and outlet are circular flow channels. Their sizes are larger than the rectangular channel, which is conducive to reducing the occurrence of blockages. The liquid enters the rectangular channel set in parallel through the flow channel inlet and flows out from the flow channel outlet after flowing through the detection unit.

## 3. Sensor Detection Principle

### 3.1. Detection Mechanism of Metal Particles

In an alternating magnetic field, changes in the electromagnetic field in the detection area occur when the metal particles pass by; this is due to the magnetization and eddy current effects. When metal particles are placed in an alternating magnetic field, their magnetization has a significant effect on the primary magnetic field. When the inductive method is used for detection, this effect is induced through the detection coil. The magnetization of metal particles in alternating magnetic fields causes a change in inductance in the detection coil and generates inductance signals. The new magnetic field generated by the magnetization increases the original magnetic field, producing an upward peak in the same direction as the original magnetic field. The eddy current reduces the original magnetic field because the new magnetic field generated by the eddy current is opposite to the original magnetic field. When the ferromagnetic metal particles pass through the detection area, the magnetization effect is greater than the eddy current effect and occupies the dominant position. The inductance value of the detection coil increases, producing an upward signal. The effect of the eddy current on non-ferromagnetic metal particles dominates and weakens the primary magnetic field. The inductance signal increases in reverse, and the signal direction is downward.

### 3.2. Magnetization Effect

Regarding the magnetization effect, our group’s previous work completed the derivation of the formula for the magnetization factor *K_p_* for metal particles:

(1) $$K_{p}=\frac{a^{3}}{2}\frac{\left(-a^{2}k^{2}+2\mu_{r}+1\right)\sin\left(ak\right)-ak\left(2\mu_{r}+1\right)\cos\left(ak\right)}{\left(a^{2}k^{2}+\mu_{r}-1\right)\sin\left(ak\right)-ak\left(\mu_{r}-1\right)\cos\left(ak\right)}$$

(2) $$k=\sqrt{-j\omega \mu_{r}\mu_{0}\sigma }$$

where a represents the radius of the particle, ω denotes the angular frequency of the AC excitation, $\mu_{r}$ signifies the relative permeability of the metal particle, and σ indicates the electrical conductivity of the metal particle [27]. It is evident from the preceding analysis that the variations in impedance due to particles moving through the coil, the alterations in inductance, and the complex magnetization coefficients of both ferromagnetic and non-ferromagnetic metal particles all exhibit intricate relationships with particle size and frequency.

An analysis of the above equation shows that the magnetization of metal particles in a time-harmonic magnetic field can be qualitatively expressed by the magnetization factor. As an important parameter of magnetization, the magnetization factor has a direct relationship with magnetic permeability, which is expressed as a complex number in the time-harmonic magnetic field, including the particle size, the magnetic field excitation frequency, the magnetic permeability of the particles, and other influencing factors. Among them, magnetic permeability exists in the form of a complex number, expressed as $\tilde{\mu }=\mu^{\prime }+j\mu^{\prime \prime }$. This represents the ability of a particular substance to respond to the magnetic field, i.e., under the applied magnetic field, how the atoms or molecules inside the substance are arranged and their sensitivity to the external magnetic field, which is a property inherent in the material of the metal particles themselves. The magnitude of permeability directly affects the magnitude and direction of the additional magnetic field generated by the magnetizing action and thus the magnitude and direction of the new magnetic field superimposed on the magnetization.

### 3.3. Eddy Current Effect

**Figure 2:**
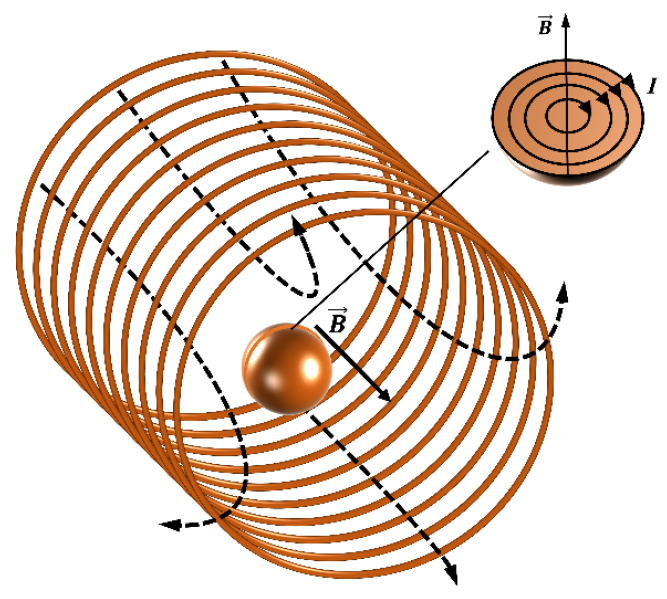
Schematic diagram of the eddy current effect.

The eddy current effect also exerts major influence on the change in the electromagnetic field of metal particles in an alternating magnetic field. According to Faraday’s law of electromagnetic induction, when a metal particle is in an alternating magnetic field, an induced electromotive force is created inside the particle, which then produces an induced current, as shown in Figure 2. According to Lenz’s law, the magnetic field created by the induced current opposes the change in the original alternating magnetic field. Therefore, the magnetic field produced by the eddy current in the metal particle is also an alternating magnetic field, but its direction is opposite to that of the original alternating magnetic field. At the same time, the presence of eddy currents also causes the metal particles to generate some of the heat; this is part of the original magnetic field of energy in the form of heat dissipation. In summary, under the influence of the eddy current effect, the electromagnetic model of metal particles in alternating magnetic fields can be divided into an inductance component and a resistance component for simulation analysis.

The equivalent circuit method is generally used to study the coupling relationship between metal particles and an alternating magnetic field. Under the action of a plane coil alternating magnetic field, only the eddy current effect of the metal particles is considered, and the metal particles can be equated to an inductive coil, so that the coupling model of the particles and the coil alternating field can be greatly simplified. Due to the mutual inductance of the metal particle-equivalent coil and the original coil, and by analyzing the equivalent inductance and the equivalent resistance of the metal particle, we can determine the impedance increment of the metal particle that arises due to the eddy current effect.

By equating the metal particle to a coil through the equivalent circuit method, mutual inductance occurs between the detection coil and the particle-equivalent coil. The coupling model of a planar coil to metal particles can be detected everywhere by means of Kirchhoff’s current and voltage laws:

(3) $$\left[\begin{array}{cc} R_{1}+j\omega L_{1} & -j\omega M\\ -j\omega M & R_{2}+j\omega L_{2} \end{array}\right]\left[\begin{array}{c} \overset{•}{I_{1}}\\ \overset{•}{I_{2}} \end{array}\right]=\left[\begin{array}{c} \overset{•}{U}\\ 0 \end{array}\right]$$

At this time, the excitation voltage of the detection coil is $\dot{U}$, the current is ${\dot{I}}_{1}$, the inductance is $L_{1}$, and the resistance is $R_{1}$; the current through the particle-equivalent coil is ${\dot{I}}_{2}$, the equivalent inductance is $L_{2}$, the equivalent resistance is $R_{2}$ and the mutual inductance of the detection coil and the particle-equivalent coil is M.

By solving the above system of equations, an expression for the coil current can be obtained, which in turn gives the impedance of the coil containing the metal particles:

(4) $$Z=\frac{\overset{•}{U}}{\overset{•}{I_{1}}}=R_{1}+\frac{\omega^{2}M^{2}}{R_{2}^{2}+\omega^{2}L_{2}^{2}}R_{2}+j\omega [L_{1}-\frac{\omega^{2}M^{2}}{R_{2}^{2}+\omega^{2}L_{2}^{2}}L_{2}]$$

Based on Equation (3), the coil equivalent inductance and equivalent resistance of the metal particles as they pass through the detection coil region are as follows:

(5) $$L=L_{1}-\frac{\omega^{2}M^{2}}{R_{2}^{2}+\omega^{2}L_{2}^{2}}L_{2}=L_{1}-\frac{M^{2}L_{2}}{\frac{R_{2}^{2}}{\omega^{2}}+L_{2}^{2}}$$

(6) $$R=R_{1}+\frac{\omega^{2}M^{2}}{R_{2}^{2}+\omega^{2}L_{2}^{2}}R_{2}=R_{1}+\frac{M^{2}R_{2}}{\frac{R_{2}^{2}}{\omega^{2}}+L_{2}^{2}}$$

According to Equations (4) and (5), the eddy currents generated by the conductive metal particles under the alternating magnetic field cause the equivalent inductance of the coil to decrease and the equivalent resistance to increase.

## 4. Simulation

**Figure 3:**
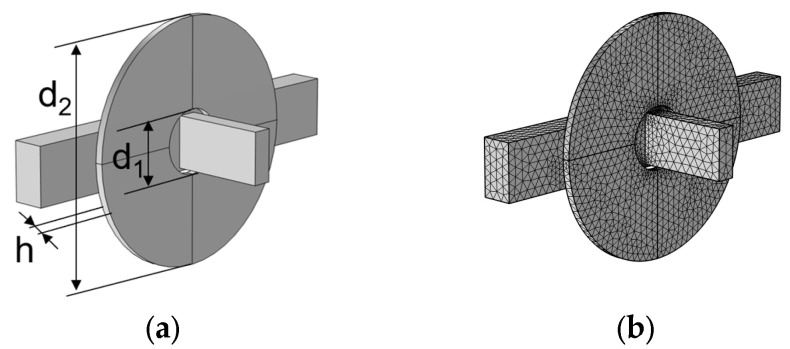
(**a**) 3D model of the sensor detection unit and (**b**) grid of the sensor detection unit.

As the source of the sensor magnetic field and the receiver of the metal particle signal, the planar coil plays a crucial role in the sensitivity and accuracy of the sensor. An analysis should take into account the influence of coil parameters, including the number of turns, diameter, and thickness on the detection signal. These parameters change the signal when metal particles pass through the detection area by affecting the magnetic field of the detection area. Therefore, we used COMSOL 6.0 simulation software to study the influence rule of the above parameters of the plane detection coil on the magnetic field of the detection area. First, COMSOL 6.0 was used to model the sensor detection unit, as shown in Figure 3, and then the model was divided into grids. The simulation model includes a flow channel, a planar coil, and a magnetic core, and their relative positions are shown in Figure 3. The magnetic core and the planar coil are placed at one side of the flow channel, and the magnetic core is placed at the center of the planar coil. d_1_ denotes the inner diameter of the coil, d_2_ denotes the outer diameter of the coil, and h denotes the thickness of the coil. The excitation mode of the planar coil adopts a constant voltage circuit with a frequency of 2 MHz and a voltage of 2 V. In addition, it should be noted that the simulation model of the coil is a numerical coil, so different turns can be set in COMSOL without changing the coil thickness.

### 4.1. Influence of the Number of Turns of the Plane Detection Coil on the Magnetic Field Distribution

**Figure 4:**
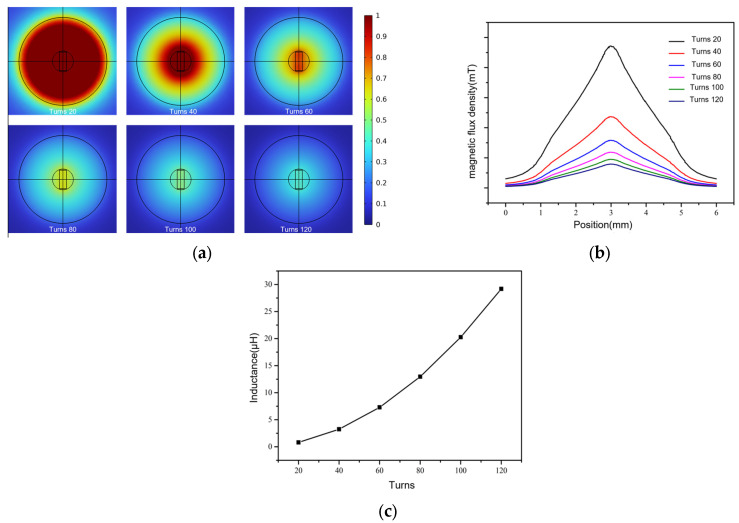
(**a**) Magnetic induction intensity distribution in the coil with magnetic cores of different lengths. (**b**) Comparison of flux density in the direction of channel length under different coil turns. (**c**) The change law for the coil inductance value with the size of the coil turns.

The control variable method was adopted to study the number of turns of the planar coil. The inner diameter of the coil was set to 0.9 mm, the outer diameter to 3.8 mm, the thickness to 0.24 mm, the excitation current frequency to 2 MHz, and the thickness and width of the magnetic core to 300 µm and 800 µm, respectively. With a length of 1.5 mm, the rectangular channel passes through the middle of the coil, directly against the core and against the coil, with a width of 500 µm and a height of 900 µm. We used 20, 40, 60, 80, 100 and 120 turns for parametric scanning to obtain the magnetic flux density distribution of the detection area, as shown in Figure 4a.

Figure 4a shows that, as the number of turns of the plane coil increases, the overall magnetic flux density of the detection area gradually decreases; the magnetic flux density is the largest at 20 turns at the center of the coil. When the number of turns increases to 40, the magnetic flux density at the edge of the coil decreases, becoming significantly weaker than that at the center. The influence of turns on the magnetic field distribution in the detection area was further studied, and the distribution curve of magnetic flux density was calculated along the length direction of the flow channel at the bottom center of the flow channel, as shown in Figure 4b.

It is clear from Figure 4b that the magnetic flux density increases first and then decreases along the length of the flow channel, reaching its maximum value in the middle of the plane coil. Compared with the magnetic flux density curves of different turns, the magnetic flux density decreases significantly after the number of turns increases, from 2.4 mT when the number of turns is 20 to 0.3 mT when the number of turns is 120. The decrease in the magnetic flux density presents a nonlinear change, and the decrease in amplitude becomes gradually smaller.

The maximum value of coil inductance falls in the range of 20 to 120 turns when making the curve of coil inductance change with the number of turns, as shown in Figure 4c. The analysis curve shows that, with the increase in the number of turns, the inductance of the coil gradually increases, from 0.2 μH at 20 turns to 27 μH at 120 turns. The change in inductance with the number of turns is the opposite to the change in the magnetic induction intensity with the coil. Therefore, the inductance value should be taken into account, while ensuring that a high magnetic induction intensity is maintained; after comprehensive consideration, 60 turns is selected as the optimal number of turns.

### 4.2. Influence of the Geometric Parameters of the Planar Detection Coil on the Magnetic Field

#### 4.2.1. Coil Inner Diameter

When making a planar-coil-type metal particle detection sensor, the flow channel is arranged in the central hole area of the coil. This is because the direction of the magnetic inductance line conforms to the right-hand rule, so the magnetic inductance line passes wholly through the hollow area; the magnetic induction intensity of the area is theoretically higher and thus more conducive to the detection of metal particles. Therefore, it is important to study the magnetic field distribution with different coil inner diameters. In order to study the influence of the inner diameter of the coil on the magnetic field distribution in the detection area, the number of turns of the coil is set to 60, the outer diameter of the coil to 3.8 mm, and the coil inner diameter to 0.9 mm, 1.0 mm, 1.1 mm, 1.2 mm, 1.3 mm, and 1.4 mm. Parametric scanning was performed on the detection area to obtain the distribution of the magnetic flux density.

**Figure 5:**
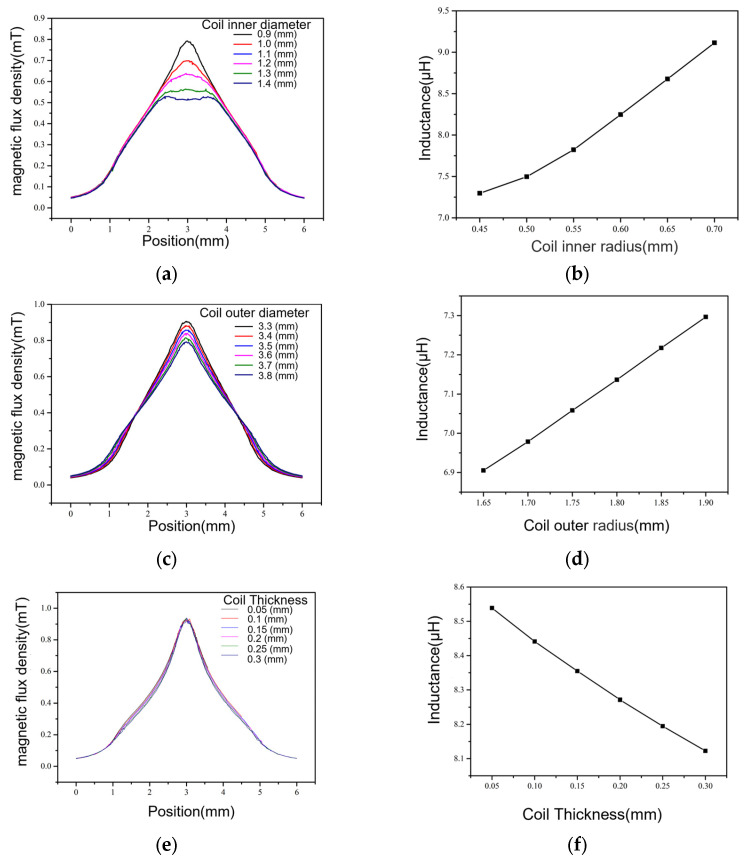
(**a**) Comparison of flux density in the direction of channel length for different inner diameters of the coil. (**b**) Variation in coil inductance with the size of the coil’s inner diameter. (**c**) Comparison of flux density in the direction of channel length for different coil outer diameter sizes. (**d**) Variation in the coil inductance value with the coil outer diameter size. (**e**) Comparison of flux density in the direction of channel length under different coil thicknesses. (**f**) Variation in coil inductance with coil thickness.

We further studied the influence of the coil’s inner diameter on the magnetic field distribution. Along the direction of flow path length, magnetic flux density distribution curves under different coil inner diameters were calculated at the bottom center of the flow path, as shown in Figure 5a.

We compared the magnetic flux density under different coil inner diameters. When the coil inner diameter is 0.9 mm, the single-peak phenomenon is more obvious, and the maximum magnetic flux density is 0.82 mT; when the coil inner diameter is 1.4 mm, the maximum magnetic flux density decreases to 0.51 mT. As the inner diameter of the coil increases, the single-peak phenomenon gradually weakens. When the diameter reaches 1.4 mm, a double-peak phenomenon appears, which is not conducive to the detection of metal particles. When optimizing the coil design, due to the high requirements for the flow channel flux, a planar coil with a larger inner diameter should be used whenever possible. When considering the flux, the double-peak phenomenon caused by the excessively large inner diameter of the coil should be avoided.

The variation curve of the maximum inductance of the coil with the coil inner diameter is obtained using statistical methods, as shown in Figure 5b. The inductance value of the coil increases with the increase in the coil inner diameter, from 7.3 μH at a diameter of 0.9 mm to 9.2 μH at a diameter of 1.4 mm. For the detection of metal particles, the enhancement of the metal particle signal mainly depends on the magnetic induction intensity of the detection area, and it is less strongly affected by the inductance value of the coil.

#### 4.2.2. Coil Outer Diameter

We studied the influence of the outer diameter of the coil on the magnetic field distribution in the detection area. The number of turns of the coil was set to 60, and the coil inner diameter was set to 0.9 mm. The coil outer diameter was set to 3.3 mm, 3.4 mm, 3.5 mm, 3.6 mm, 3.7 mm, and 3.8 mm. Parametric scanning was performed on the detection area to obtain the distribution of the magnetic flux density.

We then produced a curve of the changes in the flux density with positions at the bottom center of the flow channel length direction under different coil outer diameters, as shown in Figure 5c. Figure 5c shows that the magnetic flux density distribution along the length of the flow channel still shows, on the whole, an obvious unimodal shape. The magnetic induction intensity is small on both sides of the coil, and the magnetic induction intensity is the largest at the center. With the increase in the outer diameter of the coil, the maximum magnetic flux density in the middle position decreases from 0.9 mT at the outer diameter of 3.3 mm to 0.7 mT at the outer diameter of 3.8 mm. The larger the outer diameter, the smaller the magnetic flux density in the middle, but the larger it is on both sides. The outer diameter of the coil should be minimized. Under the condition of the same number of turns, the coil should be wound more compactly. The outer diameter and inner diameter should be as small as possible to increase the magnetic induction intensity.

The maximum inductance of the coil varies with the outer diameter of the coil, and the curve of the maximum inductance of the coil varies with the outer diameter, as shown in Figure 5d. The inductance of the coil increases with the increase in the coil outer diameter, from 6.9 μH when the outer diameter is 3.3 mm to 7.28 μH when the inner diameter is 3.8 mm. By comparing the influence of the inner diameter of the coil on the inductance value, it was found that the outer diameter of the coil has little influence on the inductance value.

#### 4.2.3. Thickness of Coil

Figure 5e indicates that the thickness of the planar detection coil has little influence on the magnetic field strength in the flow channel region. In the middle of the coil, the magnetic flux density is higher, and the peak of the magnetic flux density remains almost the same. In the direction of the length of the flow channel, the flux density varies with the thickness of the plane detection coil in the area where the flux density is about half of the peak value.

Figure 5f shows the variation law of the maximum inductance of the coil with the thickness of the coil. The peak value of the inductance of the coil decreases with the increase in the thickness of the coil in the range of 0.05–0.30 mm.

### 4.3. Influence of the Electromagnetic Parameters of the Magnetic Core on the Magnetic Field Distribution

The improvement in sensor performance by a magnetic core made of high-permeability magnetic materials largely depends on the changes in the spatial distribution of the magnetic field in the detection area. Therefore, in the process of optimizing the design of the magnetic core, it is necessary to conduct qualitative and quantitative analyses of the influence of different magnetic core materials on the magnetic field distribution. For alternating electromagnetic fields, the size of the magnetic flux density is an intuitive representation of the magnetic field distribution. Through simulation modeling and analysis, the parametric scanning of the magnetic core parameters and an analysis of the magnetic flux density distribution under different conductivity and relative permeability can effectively guide the selection of magnetic core materials. Magnetic cores of different materials can be collectively referred to as magnetizing media in electromagnetic fields. In this section, the virtual simulation software COMSOL is used to conduct the parametric scanning of the electrical conductivity and relative permeability of the magnetizing media to study the rules of their influence on the magnetic field distribution. According to the influence law obtained from the simulation results, we selected the high-permeability magnetic core material developed for the planar coil particle detection sensor.

**Figure 6:**
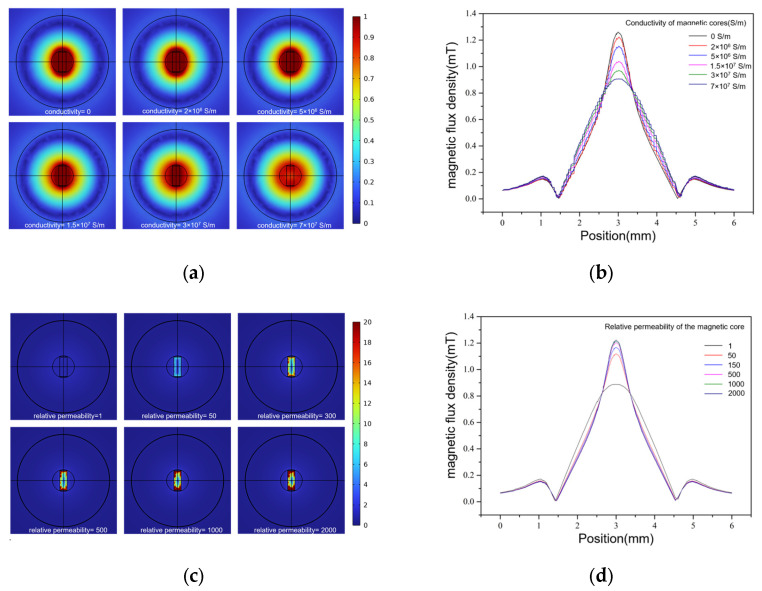
(**a**) Spatial magnetic field distribution of a single planar coil in a magnetic core with different conductivity levels. (**b**) Magnetic induction intensity distribution in the detection area under different conductivity levels in the magnetic core. (**c**) Spatial magnetic field distribution of a single planar coil in a magnetic core with different relative permeability levels. (**d**) Magnetic induction intensity distribution in the detection area under different relative permeability levels of the magnetic core.

In the simulation analysis of the conductivity of the magnetic core, the sensor model is selected as a single plane coil model; the number of turns of the plane coil is set to 60, the inner diameter is 900 µm, the thickness of the magnetic core is 300 µm, and the width is 800 µm. With a length of 1.5 mm, the rectangular channel passes through the middle of the coil, directly against the core and against the coil, with a width of 500 µm and a height of 900 µm. The relative permeability of the magnetic core is set to 2000, and its conductivity is set to: 0 S/m, 5 × 10^6^ S/m, 1.5 × 10^7^ S/m, 3.0 × 10^7^ S/m, 5.0 × 10^7^ S/m, and 7.0 × 10^7^ S/m. The coil and other parameters of the magnetic core were kept unchanged, and parametric scanning was performed. At the same time, the magnetic flux density distribution at the middle position at the bottom of the flow channel was calculated along the length direction of the flow channel, and the results are shown in Figure 6a. Figure 6b shows a curve diagram of the magnetic flux density distribution at the middle position at the bottom of the flow channel under different magnetic core conductivity levels.

Figure 6a shows that the increase in the conductivity of the magnetic core weakens the magnetic induction strength of the detection area on the whole. The higher the conductivity of the magnetic core, the stronger the eddy current effect. Therefore, the induced magnetic field generated by magnetization is weakened, resulting in the small impedance signal of the particles. The maximum value of the magnetic flux density mode decreases from 1.2 mT at a conductivity of 0 S/m to 0.9 mT at a conductivity of 7.0 × 10^7^ S/m. Figure 6b shows that, under the action of the magnetic core, the simulation results for magnetic flux density are all of the single-peak type, and there is no double-peak phenomenon. The magnetic field distribution in the detection area outside the coverage of the magnetic core is less strongly affected by the conductivity and remains almost unchanged. Within the magnetic core’s coverage area, the magnetic flux density peaks at the center, forming a distinct single peak. With the increase in conductivity, the peak value of the magnetic flux density decreases.

The influence of the relative permeability of the magnetic core on the magnetic field distribution can also be studied using the control variable method. The coil model and other parameters were kept unchanged, and the conductivity of the magnetic core was set to 1.5 × 10^7^ S/m. The relative permeability of high-permeability materials is generally 1000–3000. To study the influence of relative permeability on the magnetic flux density, the relative permeability of magnetic core was set to 1, 50, 150, 500, 1500 and 2000, and the magnetic field simulation results are compared. Parametric scanning was performed to calculate the magnetic flux density distribution in the detection area under different relative permeability values for the magnetic core. At the same time, the magnetic flux density was measured along the flow direction at the bottom center of the flow channel and a curve was drawn, as shown in Figure 6c,d.

Figure 6c,d shows that the maximum magnetic flux density increases from 0.85 mT at a relative permeability of 1 to 1.24 mT at a relative permeability of 2000. This increase occurs because higher relative permeability correlates with better permeability properties. Consequently, the magnetic field induced by magnetization has a more pronounced effect on enhancing the magnetic field of the original coil. Moreover, the magnetic field distribution of this model does not exhibit a double-peak phenomenon under different relative permeability levels.

## 5. Detection of Metallic Contaminants in Lubricating Oils

Here, we discuss the law of influence of several parameters involved in the coil and core of the sensor on the metal particle detection signal. It is especially important to note that two parameters, the conductivity and the relative permeability of the magnetic core, have a significant influence on the accuracy of the sensor in detecting metal particles. Therefore, under realistic conditions, the reasonable selection of the core material is an important influencing factor when aiming to improve the accuracy of the sensor in detecting metal particles. The scientific investigation of the influence of the core material on the metal particle detection signal is of great significance for enhancing the performance of planar coil sensors. In this subsection, we further compare the effects of the three materials used for the magnetic core (Permalloy, silicon steel sheets, and iron core) on enhancing the magnetic field; we conduct metal particle detection experiments on the basis of theoretical and simulation analyses. Moreover, we further analyze how the magnetic core materials affect particle detection by examining changes in coil inductance as the metal particles move through the detection area. The enhancement effect of the three highly permeable magnetic materials on the sensor is analyzed in detail.

### 5.1. Preparation of the Experiment

The metal particle detection system is shown in Figure 6. The system includes a micro-injection pump (Harvard pump 11 plus) as a power unit to drive the flow of oil. A magnetic core planar coil sensor is used as a sensing unit to detect oil contaminants. A microscope (Nikon AZ100, Tokyo, Japan) is used as an observation unit to observe the contaminants in the channel in real time. An impedance analyzer (Agilent E4980A, Santa Clara, CA, USA) functions as an acquisition unit to apply the excitation and read the data in real time. We use a computer with data processing functions, such as LabVIEW 2018 and MATLAB 2018, as a processing unit to display and analyze the data.

The sensor consists of detection coil wires and a magnetic core, molded using PDMS. The dimensions are 10 cm long, 5 cm wide, and 1 cm high. The channel has a diameter of 0.9 mm, with an inlet and an outlet molded at both ends. In the sensor, two planar coils and core structures with identical parameters are arranged along the channel. The two coils are connected in series to minimize the mutual inductance generated by the two coils, with a 5 cm gap between the two coils.

**Figure 7:**
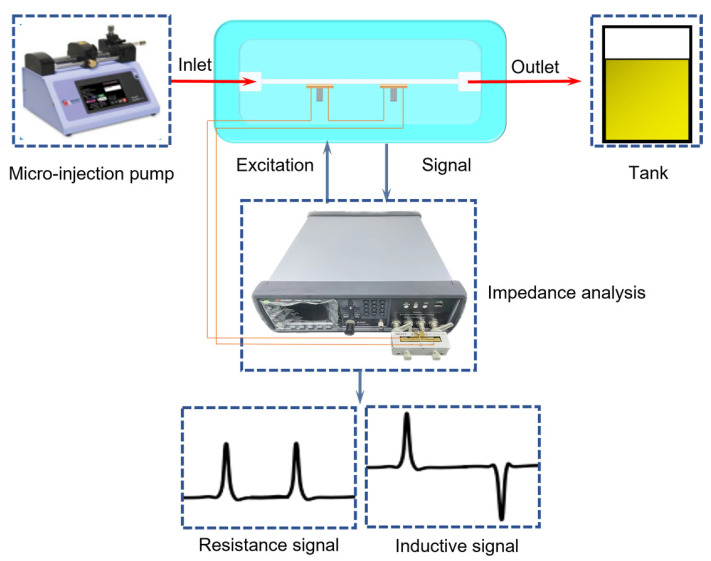
Oil metal particle detection system.

For the metal particle detection system shown in Figure 7, the oil enters the detection sensor through the micro-injection pump. Under the excitation of 2 MHz and a 2 v alternating current, when metal particles pass by, the planar coil generates resistance and an inductance pulse signals. Copper particles produce a downward inductance signal and an upward resistance signal. This is due to the eddy current effect. The reverse magnetic field is generated inside the particles, which counteracts the magnetic flux generated by the coil, thus reducing the self-inductance of the planar coil and affecting the impedance characteristics of the coil. When iron particles pass by, they generate an upward inductance signal and an upward resistance signal. This is because the magnetization effect produces the same magnetic field and increases the magnetic flux of the coil, leading to an increase in the self-inductance of the coil and affecting its impedance characteristics.

The specific process is as follows: first, a syringe (1 mL, 2 mL, 5 mL, or 10 mL) is used to extract the oil sample, and then the syringe is mounted on the micro syringe pump and the flow rate of the micro syringe pump is set. The sensor terminals are then connected to the output of the impedance analyzer as required, and the excitation parameters are set to apply AC excitation to the detection unit and measure the impedance change in real time. The frequency is set to 2 MHz and the voltage is set to 2 V. Then, the micro syringe pump is activated and a microscope is used to observe the flow of oil in the flow channel during the detection process. Particles of a specific size are selected for the experiment (the size measurement error is less than 3 μm), and at the same time the direction of the syringe pump is adjusted to make the particles move back and forth in the detection area, so as to eliminate errors caused by the characteristics of the particles themselves (such as shape, density distribution, etc.). The impedance analyzer transmits the collected impedance data, such as inductance, resistance, capacitance, etc., to the computer, and the LabVIEW program is used to display the impedance data in real time in the form of a waveform signal graph. The external environment and the detection instrument itself affect the detection results, resulting in phenomena such as signal baseline drift and noise fluctuations. For this reason, we use interpolation fitting methods to move the baseline back to zero. Finally, we use a MATLAB program to process the measured data in order to accurately determine the type, size, and quantity of particulate matter.

The sensors designed in this study are made using the mold processing method, allowing us to take full advantage of the detection area constructed by the sensing element, so that the oil contaminants are as close as possible to the coils and cores.

In order to facilitate the observations conducted by microscope in the laboratory, PDMS with good light transmission was chosen as the casting material for the sensor matrix. Liquid PDMS and coagulant were prepared in a ratio of 10:1, stirred well, and placed in a vacuum drying oven (Bonsai ZF-6020A, Beijing, China) to exclude air from the mixture. The prepared PDMS mixture was poured onto the base mold, and, when the liquid completely submerges the mold, it is then put into the vacuum drying oven to draw a vacuum and heat to solidify the PDMS. Finally, the channel mold is drawn out from the solidified PDMS to form the inspection channel, and the channel inlet and outlet molds are taken out to make the runner inlet and outlet. After completing the above four steps, the fabrication of an oil microsensor is completed. In this experiment, three types of sensors were fabricated: an iron core sensor, a silicon steel sheet sensor, and a Permalloy sensor. Permalloy is an iron–nickel alloy with excellent soft magnetic properties, and the nickel content is usually between 30% and 90%. Its relative permeability ranges from 1800 to 3000, which is higher than that of the other two materials. In this paper, we use Permalloy with a nickel content of 80–90% to provide the highest permeability. After the sensors were fabricated, 70 μm iron particles and 130 μm copper particles were selected and mixed thoroughly with the oil samples into the three sensors for detection, respectively.

### 5.2. Experimental Results and Discussion

**Figure 8:**
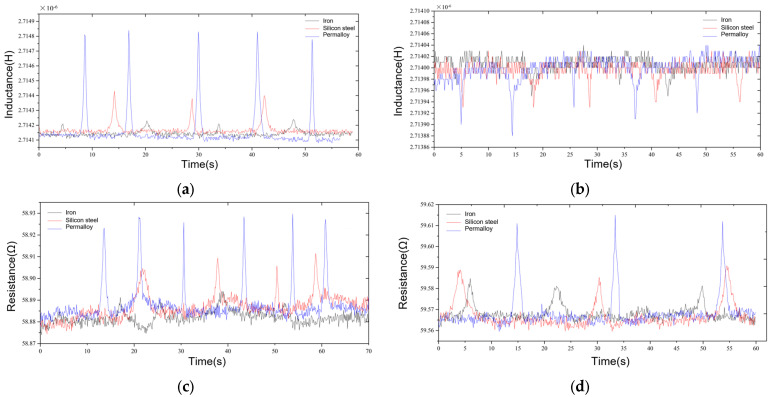
(**a**) Comparison of 70 μm iron particle inductance detection results of magnetic core sensors of different materials. (**b**) Comparison of 130 μm copper particle inductance detection results of magnetic core sensors of different materials. (**c**) Comparison of 70 μm iron particle resistance detection results of magnetic core sensors of different materials. (**d**) Comparison of 130 μm copper particle resistance detection results of magnetic core sensors of different materials.

Figure 8 shows a comparison of the inductance detection signals and resistance signals of sensors fabricated from three highly permeable material cores, i.e., the iron core, the silicon steel sheet, and Permalloy, for 70 μm iron particles and 130 μm copper particles.

The signal-to-noise ratio (SNR) is an index used to measure the signal strength relative to the background noise strength [28]. It is usually expressed in decibels (dB) and is defined as the logarithm (base 10) of the ratio of signal power to noise power. The higher the signal-to-noise ratio, the stronger the signal relative to noise and the better the communication quality. In various application fields, such as audio processing, image processing and communication systems, the signal-to-noise ratio is an important performance index. Therefore, we analyzed the detection signal amplitude and S/N ratio of copper particles and iron particles.

We compared the detection signals of the three materials of the magnetic core sensor for iron particles and copper particles. The results show that the inductive signal S/N ratio of the iron core magnetic core sensor for 70 μm iron particles is higher and reaches a value of 2.35. However, its resistance signal S/N ratio for iron particles is obviously lower than that for inductive signals, the signal amplitude is lower, and the detection effect is worse. At the same time, it performs worse for the detection of copper particles. The signal-to-noise ratio of the silicon steel sheet core sensor is improved; the signal-to-noise ratio of the inductive signal reaches 6.28 and the signal-to-noise ratio of the resistive signal reaches 6.12 for 70 μm iron particles. The signal-to-noise ratio of the inductive signal reaches 2.22 and the signal-to-noise ratio of the resistive signal reaches 3.15 for 135 μm copper particles. The signal-to-noise ratio of the inductive signal reaches 11.88, while the signal-to-noise ratio of the resistive signal reaches 8.94, which is slightly lower than the signal-to-noise ratio enhancement of the inductive signal. This is because the resistance signal of iron particles exhibits a larger fluctuation in coil resistance when no particles pass by, and the signal stability is worse than that of the inductive signal. When comparing the inductance signal and resistance signal of 135 μm copper particles, the resistance signal of the copper particles improves more obviously, and the signal-to-noise ratio reaches 10.47. Meanwhile, the signal-to-noise ratio of the inductance signal is only 3.89, which is smaller than the increase in the signal-to-noise ratio of the resistance signal. Additionally, the inductance signal of copper particles in the absence of copper particles through the signal fluctuation is larger, resulting in a lower signal-to-noise ratio. Among the three kinds of high-permeability materials, the Permalloy core sensor has the best detection effect for metal particles. Compared with the iron core material, the signal-to-noise ratio of the inductance signal of 70 μm iron particles is increased by 5.05 times, and the signal-to-noise ratio of the resistance signal of 135 μm copper particles is improved by 4.96 times. Compared with the silicon steel sheet material, the signal-to-noise ratio of the inductance signal of 70 μm iron particles is increased by 1.89 times, and the signal-to-noise ratio of the resistance signal of 135 μm copper particles is improved by 3.32 times. The signal-to-noise ratio of 70 μm iron particles is 1.89 times higher, and the resistance signal of 135 μm copper particles is 3.32 times higher. Therefore, the Permalloy core sensor can realize the efficient detection of 70 μm iron particles and 135 μm copper particles. Full width at half maximum (FWHM) refers to the width covered by a peak (such as a spectral line, waveform, curve, etc.) when it drops from its maximum value to half of its maximum value. It should be pointed out that, in Figure 8, the FWHM of the signal is relatively wide. This is because the FWHM of the particle signal is mainly related to the flow velocity of particles and oil in the flow channel [29]. In Figure 8, the flow velocity of particles and oil is about 0.01 m/s, which is slow, so there is a relatively large FWHM.

**Figure 9:**
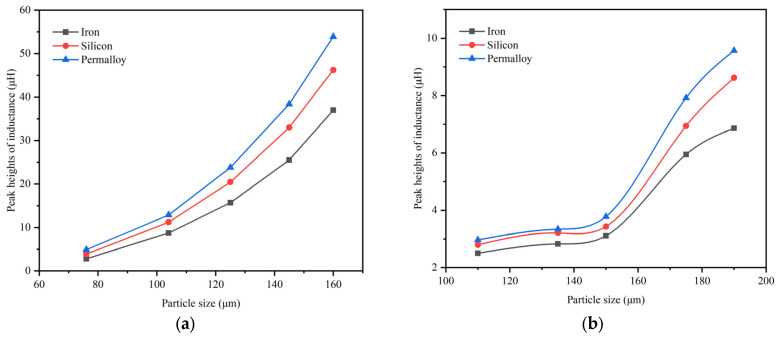
Peak heights of inductance signal with different particle size; (**a**) iron particles; (**b**) copper particles.

As shown in Figure 9, we compare the peak heights of inductance signal with different sizes for iron particles and copper particles. From Figure 9a, it can be seen that, as the diameter of the iron particles becomes larger, the peak of the inductance signal becomes larger and the slope increases. Among the three sensors, the signal detected by the permalloy core sensor is the strongest. From Figure 9b, the peak heights of the inductive signal increase as the diameter of the copper particles gets larger. However, when the diameter is between 130 and 150 μm, the speed of the signal growth becomes smaller. After 150 μm, inductive signal peak growth suddenly becomes faster.

**Figure 10:**
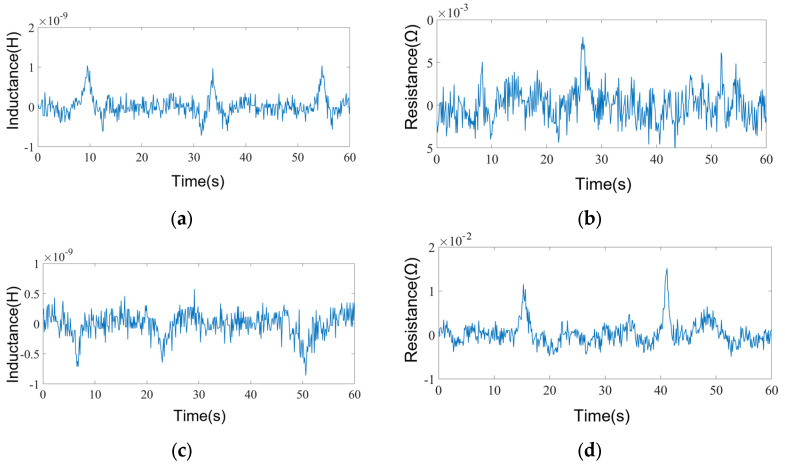
Lower detection limit of the inductance signal for (**a**) 46 µm iron particles; (**b**) 125 µm copper particles. The resistance detection floor level: (**c**) 59 µm iron particles and (**d**) 110 µm copper particles.

In order to investigate the lower detection limit of the sensor, a detection experiment for the smallest metal particles was carried out by using the Permalloy core sensor, and the detection results are shown in Figure 8. The inductance parameter can detect 46 μm iron particles and 125 μm copper particles, and the resistance parameter can detect 59 μm iron particles and 110 μm copper particles. The attributes and sizes of metal particles are determined according to the direction and presence or absence of signal pulses. By comparing the detection results of the inductance and resistance parameters, the sensors can effectively distinguish 46 µm iron particles and 110 µm copper particles. Similarly to the situation shown in Figure 8, in Figure 10, in order to study the lower detection limit, the designed flow rate is lower than 0.01 m/s, so the FWHM is larger than that in Figure 8.

## 6. Conclusions

This paper presents a method for optimizing the parameters of a sensor for detecting metal particle contaminants in oil based on the enhancement effect of high-permeability magnetic materials. The sensor uses a planar coil detection chip under the enhancement effect of a magnetic core to detect iron and copper metal particle contaminants in the oil. We conducted a finite element simulation analysis and optimized the chip structure design based on a theoretical model, exploring the impact of coil turns and aperture size on the magnetic field and coil impedance signals. We found that the planar coil performs best with 60 turns and an inner diameter of 1.2 mm; moreover, the higher the relative permeability of the magnetic core, the greater the amplitude of the inductance signal. Subsequently, experiments were conducted to verify the enhancement effect of high-permeability magnetic cores on the detection sensitivity of metal particles. A comparative analysis was performed on three types of magnetic core sensors: an iron core, a silicon steel sheet, and Permalloy. The magnetic field distribution and inductance signal changes in the sensors under the action of different high-permeability magnetic materials were investigated. The permalloy core was found to significantly improve the signal-to-noise ratio of the detection signal. Compared to the iron core material, the inductance signal-to-noise ratio for 70 μm iron particles increased by 5.05 times, and the resistance signal-to-noise ratio for 135 μm copper particles increased by 4.96 times. Compared to the silicon steel sheet material, the inductance signal-to-noise ratio for 70 μm iron particles increased by 1.89 times, and the resistance signal-to-noise ratio for 135 μm copper particles increased by 3.32 times. With a permalloy core, the planar coil sensor can detect iron particles as small as 46 μm and copper particles as small as 110 μm.

## Data Availability

The original contributions presented in the study are included in the article, further inquiries can be directed to the corresponding author.
